# *ADCY5* Mutations and Benign Hereditary Chorea

**DOI:** 10.15844/pedneurbriefs-29-9-5

**Published:** 2015-09

**Authors:** J. Gordon Millichap

**Affiliations:** 1 Division of Neurology, Ann & Robert H. Lurie Children’s Hospital of Chicago, Chicago, IL; 2 Departments of Pediatrics and Neurology, Northwestern University Feinberg School of Medicine, Chicago, IL

**Keywords:** Hereditary Chorea, Genes, Mutation Analysis

## Abstract

Investigators from the Institute of Neurology, London, UK, and centers in Italy, Germany, and Greece, studied 18 unrelated cases of benign hereditary chorea BHC (7 familial, 11 sporadic) who were negative for *NKX2-1* mutations.

Investigators from the Institute of Neurology, London, UK, and centers in Italy, Germany, and Greece, studied 18 unrelated cases of benign hereditary chorea BHC (7 familial, 11 sporadic) who were negative for *NKX2-1* mutations. The diagnosis of BHC was based on a childhood-onset movement disorder, predominantly characterized by chorea alone, with no facial myokymia. *ADCY5* and *NKX2-1* expression during brain development and in the adult human brain was assessed using microarray analysis of postmortem brain tissue. A familial case with a mild clinical presentation inherited the mutation from the affected father, whereas in a sporadic case the mutation was de novo. The nonparoxysmal generalized chorea, and dystonia in severe cases, showed significant progression of symptoms in *ADCY5* mutation carriers, in contrast to BHC secondary to *NKX2-1* mutations that showed an opposite trend, with improvement after childhood. Prominent dystonic posturing in the most severely affected *ADCY5* mutation cases of BHC is worsened by action, excitement or stress and is not a feature of the *NKX2-1* mutation cases. *ADCY5* genetic analysis should be performed in cases of benign choreiform movement disorder, even in the absence of facial myokymia. [1]

COMMENTARY. Benign hereditary chorea (BHC) is a dominantly inherited, childhood-onset hyperkinetic movement disorder characterized by non-progressive chorea and variable degrees of thyroid and respiratory involvement. Loss-of-function mutations in *NKX2-1*, a gene vital to the normal development and function of the brain, lungs, and thyroid, have been identified, leading to the description of a “brain-lung-thyroid syndrome” in some cases [2]. The present report identifies mutations in *ADCY5* as another cause of familial and sporadic BHC [1].

An extended phenotype includes obsessive-compulsive disorder and skeletal abnormalities, pes cavus and kyphosis. In a study and report from the University of Cardiff and other centers in the UK, of 10 probands with BHC and *NKX2-1* mutations, 8 presented with muscle hypotonia and 4 with hypothyroidism; only 3 of the 10 cases had the full triad of “brain-lung-thyroid syndrome [2]. An update from these UK centers highlights additional non-motor characteristics of BHC, such as cognitive impairment and psychiatric symptoms. Evidence for BHC as a developmental disorder involving impaired neural migration is discussed [3].

Investigators at Hopital Trousseau, Paris, France, report the outcome and long-term follow-up in 28 *NKX2-1* mutated BHC patients from 13 families. Chorea presenting in early infancy was associated with hypotonia, delayed walking in 25 of 28, dystonia, myoclonus and tics often associated, and ADHD occurred in 7. Among 14 patients followed until adulthood, 9 had persistent mild chorea, 2 had near total resolution of chorea but persistent myoclonus; 3 recovered completely. Learning difficulties occurred in 20/28, and 3 were mentally retarded. Various combinations of BHC, thyroid (67%) and lung (46%) features were noted. A rapid and sustained lessening of chorea was obtained in 5/8 patients treated with tetrabenazine [4].
